# CT Attenuation Values of Blood and Myocardium: Rationale for Accurate Coronary Artery Calcifications Detection with Multi-Detector CT

**DOI:** 10.1371/journal.pone.0124175

**Published:** 2015-04-14

**Authors:** Salah D. Qanadli, Anne-Marie Jouannic, Jamshid Dehmeshki, Tri-Linh Lu

**Affiliations:** 1 Department of Radiology, University Hospital of Lausanne, Rue du Bugnon 46, 1011 Lausanne, Switzerland; 2 Quantitative Medical Imaging International Institute, Digital Imaging Research center, Faculty of Computing, Information Systems and Mathematics, University of Kingston, London, United Kingdom; University of Minnesota, UNITED STATES

## Abstract

**Objectives:**

To determine inter-session and intra/inter-individual variations of the attenuations of aortic blood/myocardium with MDCT in the context of calcium scoring. To evaluate whether these variations are dependent on patients’ characteristics.

**Methods:**

Fifty-four volunteers were evaluated with calcium scoring non-enhanced CT. We measured attenuations (inter-individual variation) and standard deviations (SD, intra-individual variation) of the blood in the ascending aorta and of the myocardium of left ventricle. Every volunteer was examined twice to study the inter-session variation. The fat pad thickness at the sternum and noise (SD of air) were measured too. These values were correlated with the measured aortic/ventricular attenuations and their SDs (Pearson). Historically fixed thresholds (90 and 130 HU) were tested against different models based on attenuations of blood/ventricle.

**Results:**

The mean attenuation was 46HU (range, 17-84HU) with mean SD 23HU for the blood, and 39HU (10-82HU) with mean SD 18 HU for the myocardium. The attenuation/SD of the blood were significantly higher than those of the myocardium (p<0.01). The inter-session variation was not significant. There was a poor correlation between SD of aortic blood/ventricle with fat thickness/noise. Based on existing models, 90 HU threshold offers a confidence interval of approximately 95% and 130 HU more than 99%.

**Conclusions:**

Historical thresholds offer high confidence intervals for exclusion of aortic blood/myocardium and by the way for detecting calcifications. Nevertheless, considering the large variations of blood/myocardium CT values and the influence of patient’s characteristics, a better approach might be an adaptive threshold.

## Introduction

The quantification of coronary calcifications is now a recognized method for stratifying the risk for future cardiovascular events [1–3]. Described in the 90s by Agatston et al. (the so-called Agatston score) [4], the method has evolved using first electron beam CT technology, then dual-slice detector CT [5,6] and finally multi-detector CT [7]. One of the major requirements of the method is the adequate detection of calcifications. More refinements have been added over time to calcifications’ detection and quantification processes [8,9].

Despite new advances, the principle of the calcification scores, including the volume and the mass measurements, remains unchanged and is still based on the detection of calcified regions of interest in the vessel wall. By design, each region of interest was defined as a region satisfying two conditions: a minimum of 3 adjacent pixels (at least 1 mm^2^) with a minimum attenuation value of 130 HU [10]. Historically, this attenuation threshold had been selected to avoid hyper-attenuating pixels in the surrounding tissues (blood and myocardium) and to limit the effect of image noise induced by the acquisition protocol. The value of 130 HU, selected in the initial experience with the electron beam CT, was judged to correspond to approximately three times the attenuation of the blood and of the myocardium [4].

Introducing the helical CT technology, other authors tried to improve the sensitivity in the detection of calcification by lowering the threshold to 90 HU [6], considering the low noise level of MDCT compared to electron beam CT. However, we found no strong data supporting this threshold choice either with electron beam CT or with multi-detector CT.

The adequacy of thresholds used in calcium scoring is strongly dependent on the attenuations of blood and of myocardium, and an adequate threshold should be higher than these values. Those values may vary from volunteer to volunteer and from scan to scan. Furthermore, scan parameters may also affect the distribution of those attenuations because of inherent noise. It is therefore important to know which variation one may expect from those latter values to apprehend how they might influence calcium detection.

The objective of the current study was to determine the attenuation values of the blood and of the myocardium in the left ventricle and their distribution, using the latest-generation scanner. The secondary objective was to evaluate the influence of volunteers’ morphology and scanner parameters on these values. The third objective was to put into perspective the adequacy of the historical thresholds (i.e. 90 HU and 130 HU) used to detect calcifications, given the values and variations of blood and myocardium.

## Materials and Methods

This work was approved by the Commission of ethics and clinical research of the University of Lausanne. Written informed consent was obtained from all the participants.

### Study population

Fifty-four healthy participants with no known cardio-vascular condition were included in the study (31 men and 23 women), with a mean age of 60±12 years. Inclusion criteria: volunteers older than 18 years without the following health conditions screened during a pre-examination visit: presence of angina, active or past history of smoking ≤ 5 years, any medication for diabetes, dyslipidemia, arterial hypertension, erectile dysfunction. Technical exclusion criteria after the examination were as followed: streak artifacts due to metallic structures and motion artifacts due to breathing or arrhythmia. No volunteer was excluded.

### CT protocol

Data were acquired on a 64-detector VCT system (General Electric Medical Systems, Milwaukee, USA). Acquisitions were obtained without injection of contrast medium. All CT data were obtained with prospective ECG-gating at 75% of the R-R cycle with 120 kV and 200 mA. For each volunteer, the acquisitions were repeated twice (session 1 followed by session 2) and reconstructed with a slice thickness of 1.25 mm and with a slice thickness of 2.5 mm (54 X 2 X 2 = 216 data sets). FOV was 25 cm, and matrix was 512 X 512.

### Data analysis, measurements, and statistical analysis

The analysis was performed on an Advantage Window workstation version 4.3 (GE Medical Systems).

**Fig 1:**
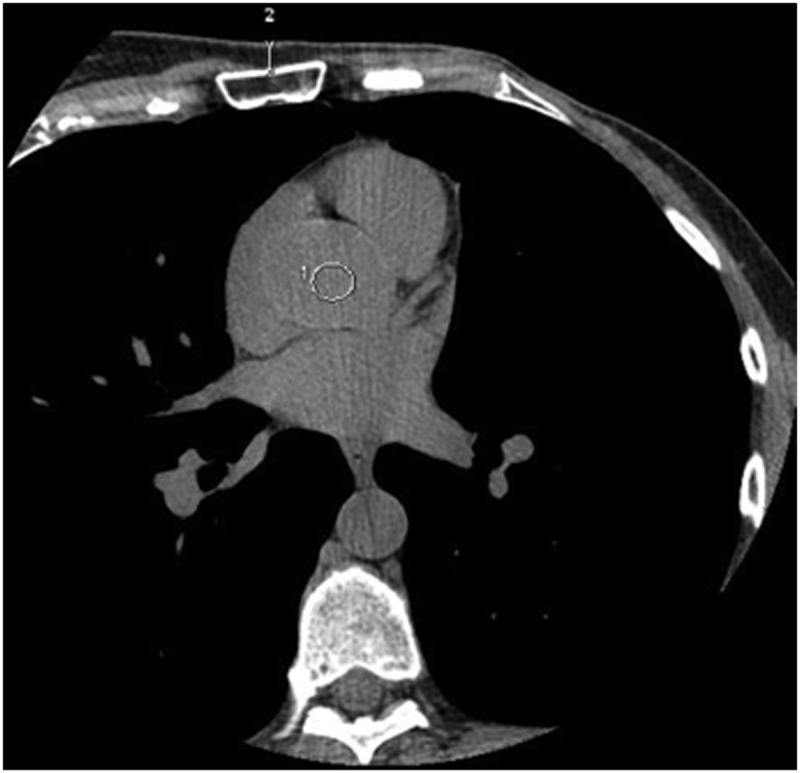
Measurements of the aortic blood attenuation and of the pre-sternal fat pad thickness, at the level of the left common coronary trunk.

**Fig 2:**
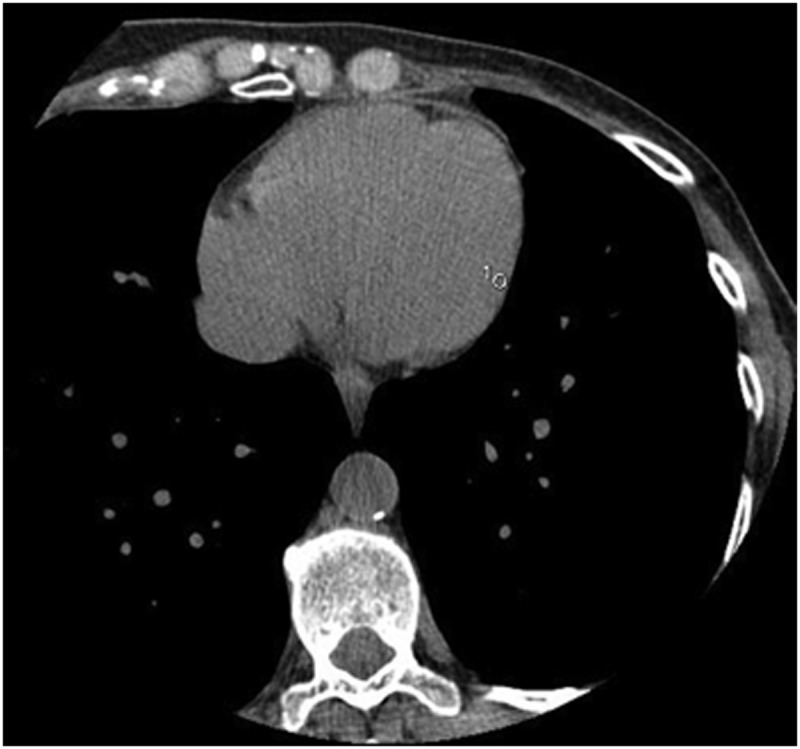
Measurement of the mid-lateral ventricular wall attenuation.

**Fig 3:**
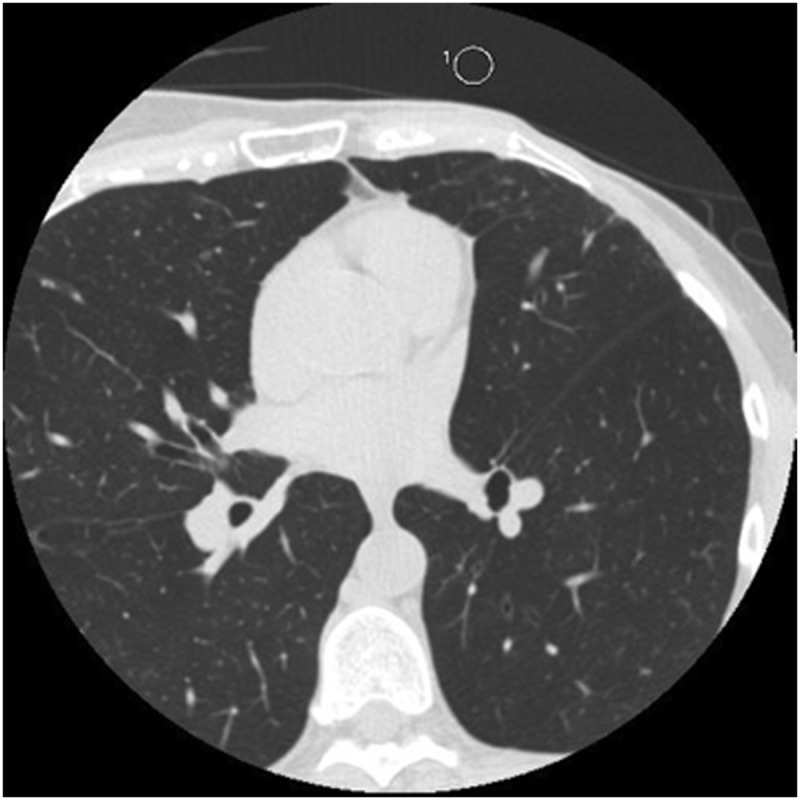
Measurement of the noise at the level of the left common trunk.

For each volunteer and for each acquisition, the following measurements were done (Figs 1 and 2): (a) the attenuation and standard deviation (SD) of the blood in the aorta at the level of the left coronary ostium (ROI of 1 cm^2^), and (b) the attenuation and SD of the medio-lateral free wall of the left ventricle (ROI of 10 mm^2^). At the same level, the pre-sternal fat pad thickness and the standard deviation of the attenuation of the air (noise) were also measured (ROI of 1 cm^2^) (Figs 1 and 3).

The mean SD in all volunteers represents the intra-individual variation. The range of values between volunteers represents the inter-individual variation. The difference of values between sessions represents the inter-session variation.

The significance of differences between measurements in one session and between measurements in two sessions were studied with a Student t-test (p<0.05). The effect of thicker slices (1.25 mm to 2.5 mm) on noise and SD of blood and myocardium was also evaluated with the t-test.

The correlation between the SDs, the fat thickness, and the noise were analyzed with a Pearson test. When the r correlation coefficient is between -1.0 and -0.7, there is a strong negative correlation. When r is between -0.7 and -0.3, the correlation is weak and negative. With a coefficient between -0.3 and 0.3, there is little or no correlation. From 0.3 to 0.7, the r coefficient shows the presence of a weak positive correlation. When the coefficient is between 0.7 and 1.0, there is a strong positive coefficient.

To evaluate the impact of the attenuations of the blood and of the myocardium on the adequacy of theoretical fixed thresholds (90 HU, 130 HU), two models were used as follows: threshold_aorta_ = HU_aorta_ + nSD_aorta_, and threshold_ventricle_ = HU_ventricle_ + nSD_ventricle_, with n = 1, 2, 3, or 4. The hypothesis is that a higher n will increase the confidence interval and will allow a better discrimination of calcifications, expecting that calcifications have higher attenuations. By definition, 1 SD represents a confidence interval of 68.2%, 2 SD 95.4%, 3 SD 99.6%, and 4 SD 99.8%. Each of these models was compared with the historical thresholds.

## Results

**Fig 4:**
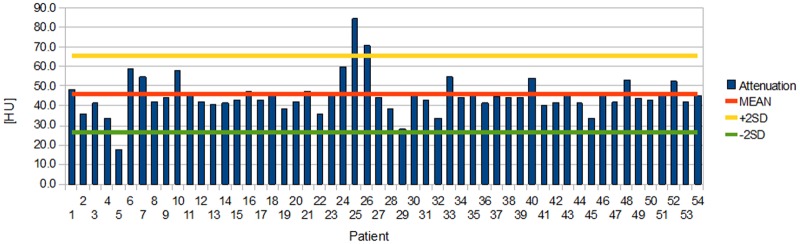
Distribution of individual aortic attenuations during the first session.

**Fig 5:**
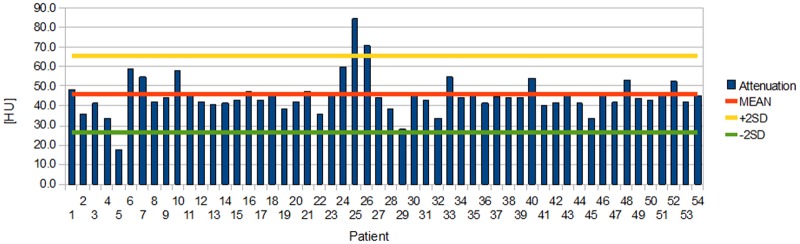
Distribution of individual ventricular attenuations during the first session.

Distributions of individual aortic and ventricular attenuations measured in the first session are presented in Figs 4 and 5 (S1 File).

In the first session, the mean attenuation of the blood in the ascending aorta was 46 HU (from 17 HU to 84 HU) with a mean SD of 23 HU. In the same session, the mean attenuation of the lateral wall of the left ventricle was 38 HU (from 16 HU to 62 HU) with a mean SD of 18 HU. The attenuation of the blood in the ascending aorta was significantly higher than the attenuation of the ventricle (p < 0.01). The standard deviation of the attenuation of the blood in the ascending aorta was also significantly higher than the SD of the attenuation of the left ventricle (p < 0.01).

In the second session, the mean attenuation of the blood in the ascending aorta was 46 HU (from 23 HU to 81 HU) with a mean SD of 23 HU. In the same session, the mean attenuation of the lateral wall of the left ventricle was 39 HU (from 10 HU to 82 HU) with a mean SD of 19HU. The attenuation of the blood in the ascending aorta was significantly higher than the attenuation of the ventricle (p < 0.01). The standard deviation of the attenuation of the blood in the ascending aorta was also significantly higher than the SD of the attenuation of the left ventricle (p < 0.01).

The intersession comparison showed that there was no difference in the attenuation of the blood in the ascending aorta and in the attenuation of the ventricle between the two sessions (p = 0.99, p = 0.44, respectively). Neither the SD of the attenuation of the blood in the ascending aorta nor the SD of the attenuation of the ventricle was different between the two sessions (p = 0.98, p = 0.08, respectively).

The averaging effect of doubling the slice thickness (from 1.25 mm to 2.5 mm) significantly reduced the image noise (19.1 HU vs 14.8 HU, p = 0.05), the SD of the attenuation of the aortic blood (25.3 HU vs 17.6 HU, p < 0.01), and the SD of the attenuation of the ventricular wall (20.0 HU vs 14.7 HU, p < 0.01). On the other hand, the measurements of the attenuation of the aortic blood and of the ventricle wall were not different when doubling the slice thickness (45.0 HU vs 46.6 HU, p = 0.26; 39.4 HU vs 37.3 HU, p = 0.25).

There was a weak positive correlation between the SD of the attenuation of the aortic blood and the fat thickness, between the SD of the attenuation of the ventricular wall and the fat thickness, between the SD of the attenuation of the aortic blood and the noise, and between the SD of the attenuation of the ventricular wall and the noise (Table 1). There was also a weak positive correlation between the fat pad thickness and the noise (r = 0.41, p < 0.01). There was no or poor correlation between the attenuation of the blood and the fat thickness and the noise (r = 0.14, p = 0.06 and r = 0.16, p = 0.04; respectively). Neither was there a correlation between the attenuation of the myocardium and the fat thickness and the noise (r = -0.13, p = 0.10 and r = -0.12, p = 0.13; respectively).

**Table 1:** Pearson correlation coefficients between adaptive thresholds, fat pad thickness and noise.

	Fat pad thickness	Noise
Threshold based on aortic blood attenuation	r = 0.48	r = 0.37
	p < 0.01	p < 0.01
Threshold based on ventricle wall attenuation	r = 0.24	r = 0.27
	p < 0.01	p < 0.01

Note—Threshold_m_ = HU_m_ + 3SD_m_, m = aortic blood or ventricle wall

When we calculated the model based on the individual attenuation of the aortic blood plus n SD (HU_aorta_ + n SD_aorta_), there was a large variation. As an example, for n = 3, the threshold values vary from 59.8 HU to 183.4 HU (mean = 113.4 HU). The model based on the attenuation of the ventricle (HU_ventricle_ + n SD_ventricle_) also showed a large variation: for n = 3, the thresholds vary from 49.8 HU to 193.8 HU (mean 93.1 HU). Table 2 presents the threshold values with one SD, two SDs, three SDs, and four SDs with the corresponding intervals of confidence. The threshold based on the attenuation of the ventricle was significantly lower than the threshold based on the attenuation of the aortic blood (p < 0.01 for n = 1, 2, 3 or 4). There was no significant difference for the two threshold methods between sessions (p = 0.99 and 0.07, respectively).

**Table 2:** Models of thresholds based on attenuation values of aortic blood and myocardium.

	Threshold based on aortic blood attenuation (HU)	Threshold based on ventricle wall attenuation (HU)	Interval of confidence (%)
1 SD	68.2	56.8	68.2
2 SD	90.8	74.9	95.4
3 SD	113.4	93.1	99.6
4 SD	136.0	111.2	99.8

Models were built on the mean attenuation value maximized by 1, 2, 3 or 4 SD.

## Discussion

First described by Agatston et al. [4], the calcium score is a fast and simple method for assessing calcium coronary deposits. Nevertheless, the calcium score has rapidly shown a deficiency in the detection and the quantification of small lesions [11]. Different authors have proposed other methods like measuring calcium volume and calcium mass to improve the detection of calcifications and the reproducibility of the measurements [8,9]. On the other hand, the question of applying an adaptive threshold to better investigate calcifications has scarcely been discussed [12,13].

In the present study, we found large variations in the measurements of attenuations in individual volunteers as well as between volunteers. In the study of Raggi et al. [12], this observation has also been made both in terms of intra-individual measurements and in terms of inter-individual measurements with electron beam CT. The authors stated that such variation may be explained by the influence of the body habitus and of the fat repartition on the quality of the images. Thus, in patients with larger body mass, photons may interact with more matter and may subsequently increase the scatter effect and thus the background noise. Although the study of Raggi et al. was done with an electron beam CT, we think that such an explanation remains true for multidetector CT [14], as low dose protocols are generally used in calcium scoring. Besides, in our study we effectively found a positive correlation between the SD of the blood / myocardium and the fat pad thickness. Furthermore, the fact that doubling the slice thickness significantly reduced the noise and the SDs of attenuation measurements underlines the presence of image degradation due to scatter effect. However, it is not clear if increasing the slice thickness will bring a benefit to the calcium score measurement. It has been shown that thinner slabs lead to higher calcium scores. But whether this score increase in thinner slices is due to the presence of more noise or due to a better detection of smaller lesions remains difficult to assess [15].

When we compare our results to the historical fixed thresholds of 90 HU and 130 HU [6,4], it can be noted that 90 HU and 130 HU correspond approximately to the aortic blood attenuation plus 2 SD or 4SD, with confidence intervals of 95.4% and 99.8%, respectively. Thus, those fixed thresholds have an observational justification. However, considering the large variation of attenuation measurements, the use of a fixed threshold is not the most precise way to evaluate coronary calcifications. An adaptive method tailored to each patient is then more suitable for such a task. This adaptive method should take into account the patient's characteristics and to the scanner’s technical parameters. Further studies will have to confirm this hypothesis.

A limitation of this study is that we did not directly evaluate the effect of varying the scanner parameters on attenuation measurements and we used the noise as a surrogate. We think that this way allows us to take into account the interfering effect of the volunteers' body habitus and to avoid exposing the volunteers to multiple scans with more radiation. Another limitation is that we did not appraise the quantitative effect of varying thresholds on the calcium score or the adequacy of an adaptive threshold. However, other authors have already studied this question and have shown the important influence of thresholds on calcium measurements [13,16]. For example, Groen et al. have shown with a phantom model that using a threshold adjusted to the HU_peak_ of calcifications allows measurements that are more accurate and less susceptible to cardiac motion [17].

## Conclusions

In conclusion, there are important intra- and inter-individual variations in terms of attenuations of the blood and of the myocardium. Although a fixed threshold of 90 HU has a confidence interval of 95.4% and a fixed threshold of 130 HU a confidence interval of 99.8%, our results have important consequences on the adequacy of these commonly used fixed thresholds for calcium detection. Moreover, the intra-individual variation seems to be dependent on patient’s characteristics and scan parameters. Therefore, an adaptive threshold may be more adequate than a fixed threshold.

## Supporting Information

S1 File Data collected from all patients for the two sessions. (PDF)
