# Supplementary material for: CT Attenuation Values of Blood and Myocardium: Rationale for Accurate Coronary Artery Calcifications Detection with Multi-Detector CT
Source: PLoS One. 2015 Apr 14;10(4):e0124175. doi: 10.1371/journal.pone.0124175 (PMC4397043; doi:10.1371/journal.pone.0124175)
Supplement: S1 File — (PDF) [file pone.0124175.s001.pdf]

Name	Age	Session		Aorta	STD	Fat thick	Noise	Slice		Ventricle	STD	Slice
AA	47	1.0		47.6	16.1	9.3	8.3	152/16		46.2	12.3	152/36
AM	44	1.0		43.6	31.5	28.3		2/38		37.4	23.6	2/78
AGM	58	1.0		41.4	25.7	8.0	17.9	2/27		22.9	12.3	2/71
AC	88	1.0		33.7	23.7	6.1	30.2	2/48		45.6	21.9	2/95
BP	55	1.0		17.4	14.2	7.1	14.2	45/2		24.6	12.4	82/2
BJ	79	1.0		52.5	24.1	26.9	18.5	172/20		46.6	16.4	172/37
CR	81	1.0		54.7	17.5	6.3	9.6	2/47		53.1	14.0	2/81
CHR	67	1		41.8	26.6	18.1	16.5	2/33		41.5	12.5	2/68
CM	61	1.0		43.9	36.1	5.9	18.7	30/2		42.8	43.3	75/2
CI	53	1.0		58.2	39.5	29.1	25.0	2/31		32.4	29.5	2/62
CS	37	1.0		45.5	15.5	13.7	9.6	1/172		21.7	17.7	37/172
CJ	47	1.0		39.3	13.8	9.5	7.4	172/18		43.2	11.1	172/43
CD	67	1.0		40.6	25.2	4.6	19.7	17/2		26.8	19.5	58/2
DP	66	1.0		41.5	39.5	25.4	29.1	28/2		19.8	37.3	68/2
DM	64	1.0		43.1	23.3	10.8	18.5	24/2		39.5	28.0	70/2
DJF	60	1.0		47.9	19.3	12.2	13.5	102/11		39.1	22.8	102/36
DY	58	1.0		46.1	24.3	25.2	16.6	2/47		26.6	17.6	2/85
EP	54	1.0		48.7	11.8	5.6	8.4	172/14		26.2	11.1	172/37
EJ	51	1.0		38.6	28.5	12.5	17.2	12/2		40.2	32.7	50/2
FM	54	1.0		41.8	22.5	3.6	14.7	2/17		29.1	13.2	2/71
FY	43	1.0		47.7	19.2	9.3	16.5	2/26		54.0	14.3	2/82
FA	32	1.0		36.0	14.3	3.2	9.2	51/2		38.3	8.0	104/2
GM	54	1.0		45.9	16.1	10.0	9.4	172/21		41.4	7.7	172/43
GP	75	1.0		59.6	26.9	14.9	17.8	2/43		53.8	18.7	2/87
GA	59	1.0		84.3	32.4	14.7	18.7	2/48		52.5	28.2	2/83
GJR	49	1.0		70.4	21.6	14.9		2/37		55.0	15.4	2/66
GN	53	1.0		44.0	28.0	5.6	17.4	2/36		34.0	17.5	2/80
LM	79	1.0		41.1	11.7	4.8	6.5	172/27		40.8	8.7	172/46
MG	56	1.0		27.8	18.6			2/52		51.4	12.1	2/86
MR	78	1.0		41.5	17.0	15.4	24.2	172/15		42.3	15.5	172/38
MF	55	1.0		43.0	26.5	8.8	17.0	2/37		31.0	19.6	2/79
MM	51	1.0		36.3	17.4	14.9	11.3	152/18		42.6	14.3	152/39
MIF	60	1.0		54.8	40.1	17.2	29.3	1/2		48.0	29.3	82/2
MA	62	1.0		44.0	35.9	18.2	20.3	2/23		39.5	31.6	2/69
MD	44	1.0		45.8	20.5	20.5	16.3	2/41		21.6	11.0	2/76
NM	54	1.0		41.4	15.6	8.1	11.2	45/2		45.9	15.2	87/2
NYM	68	1.0		44.5	32.7	11.5	23.2	2/24		33.2	21.3	2/65
PR	71	1.0		43.9	14.9	3.6	11.2	2/29		54.6	11.8	2/70
PC	63	1.0		44.3	19.6	12.6	17.2	2/33		34.3	18.7	2/58
PH	79	1.0		53.9	26.9	9.3	13.7	2/42		38.4	12.8	2/76
RA	58	1.0		40.4	31.5	18.1	19.5	2/27		51.2	23.6	2/76
RM	52	1.0		41.6	23.4	8.1	17.9	18/2		21.7	25.7	60/2
ROM	69	1.0		45.6	22.4	15.0	21.2	2/46		30.2	14.7	2/83
RS	35	1.0		44.1	18.1	12.8	17.8	1/2		27.7	28.9	34/172
SD	60	1.0		33.6	33.9	6.6	32.7	26/2		33.6	22.1	64/2
SG	79	1.0		46.3	24.1	1.7	13.3	2/70		55.9	20.0	2/102
SB	57	1.0		42.1	11.6	2.3	7.2	102/11		28.8	7.0	102/37
TM	54	1.0		52.9	27.9	5.6	21.1	21/2		29	26.7	69/2
TF	62	1.0		43.3	25.9	5.6	16.7	28/2		47.0	22.4	62/2
VA	65	1.0		43.1	24.8	8.5	17.4	2/20		26.5	16.9	2/57
VR	67	1.0		45.0	25.8	3.9	19.3	2/12		46.8	22.7	2/53
WP	72	1.0		52.3	24.7	9.3	19.0	2/49		35.4	17.0	2/82
WE	67	1.0		42.0	21.6	3.8	12.5	2/15		39.3	16.2	2/67
WJ	68	1.0		45.3	21.4	3.1	14.5	2/20		37.1	19.1	2/69
